# Diffuse Reflectance Spectroscopy of the Oral Mucosa: In Vivo Experimental Validation of the Precancerous Lesions Early Detection Possibility

**DOI:** 10.3390/diagnostics13091633

**Published:** 2023-05-05

**Authors:** Alexander V. Kolpakov, Anastasia A. Moshkova, Ekaterina V. Melikhova, Diana Yu. Sokolova, Natalia P. Muravskaya, Andrey V. Samorodov, Nina O. Kopaneva, Galina I. Lukina, Marina Ya. Abramova, Veta G. Mamatsashvili, Vadim V. Parshkov

**Affiliations:** 1 Faculty of Biomedical Engineering, Bauman Moscow State Technical University, Moscow 105005, Russia; nast.moshkova@bmstu.ru (A.A.M.); whyclos@mail.ru (D.Y.S.); muravskaya@bmstu.ru (N.P.M.); avs@bmstu.ru (A.V.S.); 2 Department of Therapeutic Dentistry and Diseases of the Oral Mucosa, Moscow State University of Medicine and Dentistry, Moscow 127473, Russia

**Keywords:** diffuse reflectance spectroscopy, cancer screening, precancerous lesion detection

## Abstract

This article is devoted to the experimental validation of the possibility of early detection of precancerous lesions in the oral mucosa in vivo using diffuse reflectance spectroscopy in the wavelength range from 360 to 1000 nm. During the study, a sample of 119 patients with precancerous lesions has been collected and analyzed. As a result of the analysis, the most informative wavelength ranges were determined, in which the maximum differences in the backscattering spectra of lesions and intact tissues were observed, methods for automatic classification of backscattering spectra of the oral mucosa were studied, sensitivity and specificity values, achievable using diffuse reflectance spectroscopy for detecting hyperkeratosis on the tongue ventrolateral mucosa surface and buccal mucosa, were evaluated. As a result of preliminary experimental studies in vivo, the possibility of automatic detection of precancerous lesions of the oral mucosa surface using diffuse reflectance spectroscopy in the wavelength range from 500 to 900 nm with an accuracy of at least 75 percent has been shown.

## 1. Introduction

According to the World Health Organization, the frequency of detection of oncological diseases localized in the oral cavity in different regions of the world is up to twenty cases per 100,000 population [1,2,3]. In the Russian Federation, the number of diseases of the oral mucosa has increased by more than 20 percent in 10 years [4]. The increase in the incidence of oral cavity oncological diseases in the Russian Federation for the period from 2009 to 2019 amounted to 35.6% [5].

Among the important factors causing pre-tumor and tumor changes in the oral mucosa are tobacco smoking and chronic mechanical injury, including those caused by prosthetics, human papillomavirus, herpes virus, etc. [6,7].

Progressive stages of inflammatory processes, also known as hyperkeratosis areas of the oral mucosa, can cause the development of malignant neoplasms [7,8,9,10,11,12,13].

Given these facts, the development of new hardware and software methods for detecting precancerous lesions of the oral mucosa at an early stage, the most favorable for treatment, is an important and socially significant task.

The primary methods for diagnosing lesions of the oral mucosa include visualization in the visible range, and immunological, histological, and fluorescent methods [14,15,16,17,18,19,20].

When visualizing in the visible wavelength range, intraoral cameras and endoscopes are used to record images of oral cavity areas in reflected radiation. The disadvantage of visualization of the visible range is the subjective nature of the detection of lesions and, as a result, the difficulty of detecting them at an early stage [20].

With the immunological method, using enzyme immunoassay analyzers, studies are performed to identify biochemical markers of the pathological process: TNF-α, IL 1–10, C-reactive protein, Ki-67, P-53, etc. [14]. However, the minimum detectable concentration of biochemical markers is already reached in the advanced stage of the pathological process, and the equipment used is expensive.

The fluorescent method is based on the registration of the luminescence of microorganisms and their metabolites caused by exposure to optical radiation and allows the detection of caries lesions [15,16], inflammation, and oncological lesions of the oral mucosa [17,18,19]. The disadvantage of the method is the subjective nature and low reproducibility of the diagnostic results. According to published data, the sensitivity of the fluorescent method varies in the range from 30% to 100%, while the specificity varies from 15% to 100% [20].

An important advantage of optical diagnostic methods, particularly backscattering spectroscopy, is their non-invasiveness and the possibility of detecting structural inhomogeneities in tissues at depth [21]. Several laboratories have shown the effectiveness of the diffuse reflectance spectroscopy (hereinafter—DRS) method for detecting optical inhomogeneities, including oncological neoplasms in biological tissues, both ex vivo and in vivo [22,23,24,25,26,27,28,29,30,31,32,33,34,35,36,37,38,39,40,41,42,43,44,45,46,47,48]. In particular, the effectiveness of infrared DRS for the detection of precancerous lesions in oral mucosal tissues in vitro has been shown based on the results of a study of oral fluids [29,30]. In [45], an experiment on non-biological models showed that the error in determining the optical parameters by DRS does not exceed 10%.

Histological examination of oral tissue samples obtained through biopsy is the most reliable method for diagnosing cancers, but it is characterized by such disadvantages as invasiveness and dependence of diagnostic results on the doctor’s qualifications [20]. Currently, the immunological method in combination with histological examination is the gold standard for diagnosing precancerous lesions of the oral mucosa [43,44].

Several researchers have shown the effectiveness of machine learning methods and automatic classification of spectral data in detecting cancerous lesions [31,37,46]. For example, in [31], the effectiveness of Raman spectroscopy in detecting cancerous lesions of the oral cavity was studied. The models considered in this paper showed an accuracy of classification of cancerous lesions of 81.25% when using the principal component method and linear discriminant analysis and 87.5% when using the principal component method and quadratic discriminant analysis. As a result of terahertz laser spectroscopy approbation on oral fluid samples in vitro, the possibility of detecting lichen planus according to the results of the classification of spectral data with an accuracy of more than 80% was shown [37].

Studies of the DRS in vivo in the wavelength range from 450 to 650 nm have shown the effectiveness of this method for detecting oncological areas in the intestinal mucosa [35]. Authors of the paper [36] published the results of a study of in vivo DRS in the wavelength range from 500 to 750 nm in combination with endoscopic imaging and autofluorescence registration to detect areas of dysplasia in the epithelium of the oral mucosa. As a result of this study on 13 volunteers, it was found that with the development of dysplasia in the oral mucosa, the reflection coefficient of tissues in the wavelength ranges from 600 to 750 nm increases; however, no diagnostic criteria have been established for the automated detection of foci of dysplasia and data on the diagnostic characteristics of the method are not presented. Additionally, published studies do not provide information on changes in the optical properties of tissues with the development of pathology in the oral mucosa at wavelengths above 750 nm. At the same time, it is advisable to study the dynamics of optical properties during the development of precancerous lesions of the oral mucosa also in the wavelength range from 360 to 450 nm, in which the fluorescence of mucosal tissues is registered [17,19,41], as well as in the near-infrared range wavelengths from 750 to 1000 nm, in which the absorption maxima of tissue chromophores (hemoglobin and water) and the dynamics of the scattering properties of biological tissues are recorded [21].

The aim of this study was to determine the possibility of detecting precancerous lesions of the oral mucosa in vivo using DRS in the wavelength range from 360 to 1000 nm. To achieve the goal, it is necessary to solve the following tasks: to determine the most informative wavelength ranges in which there are maximum differences in the backscattering spectra of the lesion and intact tissue areas; to develop a method for automatic classification of backscattering spectra of the oral mucosa; and to determine the sensitivity and specificity values for the detection of precancerous lesions using DRS.

This article presents the preliminary results of studies conducted on a sample of in vivo registered backscatter spectra of hyperkeratosis areas on the oral mucous membranes.

## 2. Materials and Methods

We conducted the study on 119 patients who underwent a clinical examination at the clinic of the Moscow State University of Medicine and Dentistry. The study protocol was approved by the Interuniversity Ethics Committee (Protocol No. 10 dated 25 November 2021). Voluntary informed consent for the study was obtained from all patients who participated in the study.

We registered backscattering spectra of oral tissues using a Thorlabs (Newton, MA, USA) SLS201L broadband source generating radiation in the wavelength range from 360 to 2600 nm [25]. The radiation was delivered to the studied tissue area by a fiber optic probe. We used an Avantes AvaSpec-2048 (Apeldroon, The Netherlands) spectrometer with an installed fiber optic sensor for backscattered radiation registration. Spectrometer AvaSpec-2048 measured radiation intensity in the wavelength range from 325 to 1100 nm.

In order to verify the metrological characteristics of the AvaSpec-2048, we compared the measurement errors of AvaSpec-2048 and Thorlabs CCS200 spectrometers. Thorlabs CCS200 (Serial number M00785262) spectrometer was calibrated by equipment with confirmed metrological traceability to national standards of state participants “EURAMET” (NPL, PTB, BNM, etc.) and “NIST” (USA) (Calibration certificate dated 29 October 2021). To compare the measurement errors of two spectrometers, a series of 30 measurements of the emission spectrum of the Thorlabs SLS201L source was performed, and the data is presented in the manufacturer’s documentation [32]. To estimate the measurement error, we used the normalized value of the standard deviation (RMS) of measurements, calculated from a series of 30 measurements of the Thorlabs SLS201L spectrum in the wavelength range from 360 to 1025 nm. The obtained values of the normalized RMS measurements for the Thorlabs CCS200 spectrometer turned out to be in the range of values from 0.02 to 0.01, and for the AvaSpec-2048 spectrometer, they were in the range of values from 0.002 to 0.001. Thus, the measurement errors of the AvaSpec-2048 are an order of magnitude smaller than the measurement errors of the Thorlabs CCS200. We can conclude that the measurement results of the AvaSpec-2048 are sufficiently accurate and that they can be used in this study. The registered spectra of oral mucosal sections were transferred from the spectrometer via a USB connection to a PC for further processing and analysis.

During the first stage of the study, we registered the spectra samples of 14 areas of hyperkeratosis and eight intact non-keratinized areas of the mucous membrane on the ventrolateral surface of the tongue, as well as 6 areas of hyperkeratosis and seven intact areas of the mucosa localized on the buccal mucosa.

**Figure 1:**
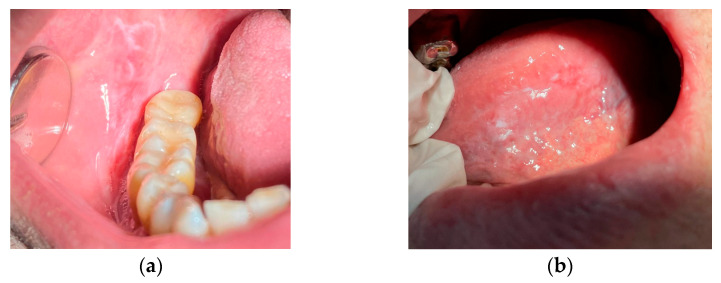
Examples of images of the lesion areas of the oral cavity: an area of hyperkeratosis on the surface of the buccal mucosa (**a**) and on the ventrolateral surface of the tongue (**b**).

Examples of images of lesions are shown in Figure 1.

We registered a series of 15 to 30 backscattering spectra for each studied area of the oral cavity. The average normalized spectrum for each area was calculated from each series:

(1) $$I_{n}\left(\lambda \right)=\frac{I\left(\lambda \right)}{I_{source}\left(\lambda \right)},$$

where $I\left(\lambda \right)$—the average normalized spectrum of the area,

$I_{source}\left(\lambda \right)$—the average normalized spectrum of the light source.

The averaged normalized spectra of each section were further used to study classification algorithms. For each sample of the averaged spectrum, a 95% confidence interval was calculated:

(2) $$E\left(\lambda \right)=I\left(\lambda \right)\pm t_{1-\frac{\alpha }{2},n-1}\frac{S_{e}\left(\lambda \right)}{\sqrt{n}},$$

where n—the sample size of the recorded spectra of the area,

$t_{1-\frac{\alpha }{2},n-1}$—α-quantile of Student’s distribution (α = 0.05) c n−1 degrees of freedom.

**Figure 2:**
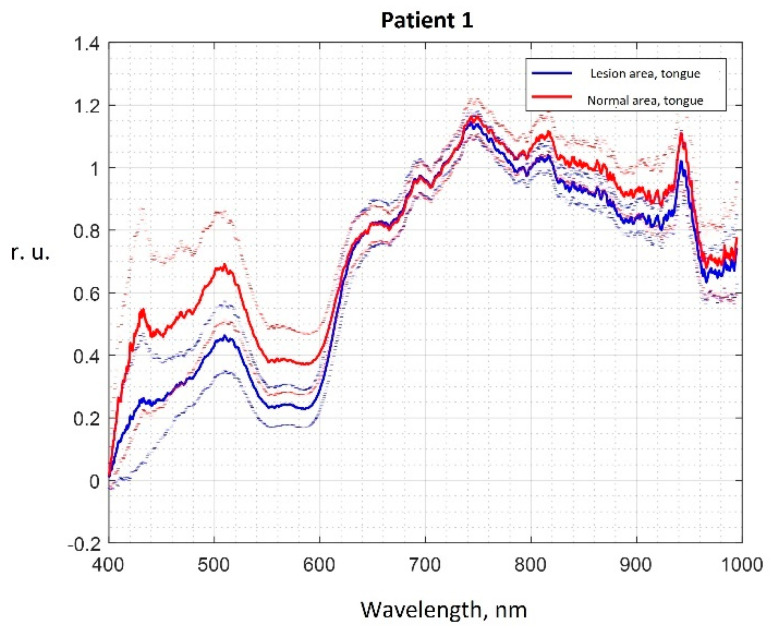
Graphs of the averaged spectrum of the area of hyperkeratosis (blue) and the intact area on the tongue (red) in patient No. 1. The solid line is the averaged spectrum, the dashed lines are the boundaries of the confidence intervals.

Examples of averaged normalized spectra of areas of the oral mucosa in relative units (r.u.) with boundaries of 95 percent confidence intervals are shown in Figure 2.

Changes in the optical properties of oral mucosa tissues in hyperkeratosis are explained by changes in the intensity of mucous membrane tissues fluorescence [17,18,19,41], changes in the concentration of chromophores in tissues, the main of which are oxy- and deoxyhemoglobin, water, as well as changes in the scattering properties due to the growth of the epithelium layer during keratinization [21,33,42]. Lower intensities of the spectra in the wavelength range from 400 to 600 nm in the lesioned areas correspond to higher concentrations of oxy-, deoxyhemoglobin, and water, caused by, in particular, the higher number of microvessels, which correlates with a higher number of mast cells during premalignant epithelial lesions progression [49]. Visually observed differences in the averaged spectra of hyperkeratosis and intact areas in the wavelength range from 400 to 600 nm correspond to a decrease in the intensity of tissue fluorescence in this lesion, which is also observed in studies of other authors [41]. Differences in spectra in the range from 800 to 1000 nm (Figure 2) correspond to a change in the scattering properties of epithelial tissues in the near IR wavelength range during the growth of the stratum corneum [21,33,42] and an increased number of mast cells during inflammation and premalignant lesions progression in oral mucosa [49,50].

To select the most informative sub-ranges of lengths suitable for further construction of an automatic classifier, we used the method of one measurement projection [34]. According to this method, the measure of the difference between the spectra of intact and lesioned areas at wavelength λ is coefficient Q, which is calculated by the formula:

(3) $$Q\left(\lambda \right)=\frac{I_{norm}\left(\lambda \right)-I_{les}\left(\lambda \right)}{\sqrt{\sigma {\left(\lambda \right)}_{norm}^{2}+\sigma {\left(\lambda \right)}_{les}^{2}}},$$

where $I_{norm}\left(\lambda \right),I_{les}\left(\lambda \right)$—the value of the average spectrum at a given λ for the intact and lesioned area, respectively;

$\sigma {\left(\lambda \right)}_{norm}^{2},\sigma {\left(\lambda \right)}_{les}^{2}$—sample variance of the average spectrum value at a given wavelength λ for the intact and lesioned area, respectively.

**Figure 3:**
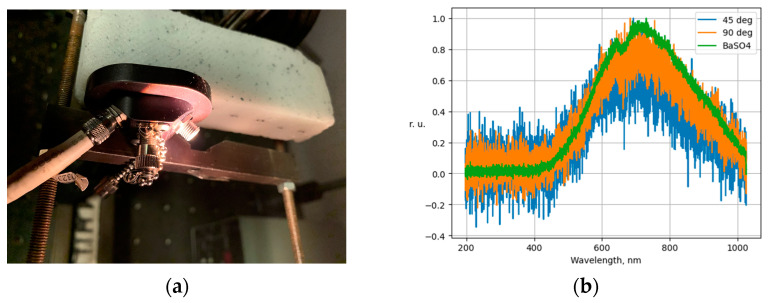
Scheme of the test object spectra registration (**a**); spectra of the test object at different angles of the radiation source and sensor (**b**).

As a result of experimental testing on a stand using a test object, it was found that the proposed registration and processing scheme ensures the invariance of the registered spectra with respect to the angles of the fiber optic source and sensor. During the experiment, a series of spectra of the BaSO4 reflector were registered using the spectrometer Thorlabs CCS200, as well as a series of spectra of the test object at relative angles of the source and receiver of radiation of 45 and 90 degrees. The angle of the location of the radiation source and sensor was controlled using a mechanical lock (Figure 3a). The spectra of the test object at different angles of the source and receiver of radiation are shown in Figure 3b.

As a result of calculating the Q coefficient by Formula (3), it was found that the spectra registered at different angles did not have statistically significant differences.

**Figure 4:**
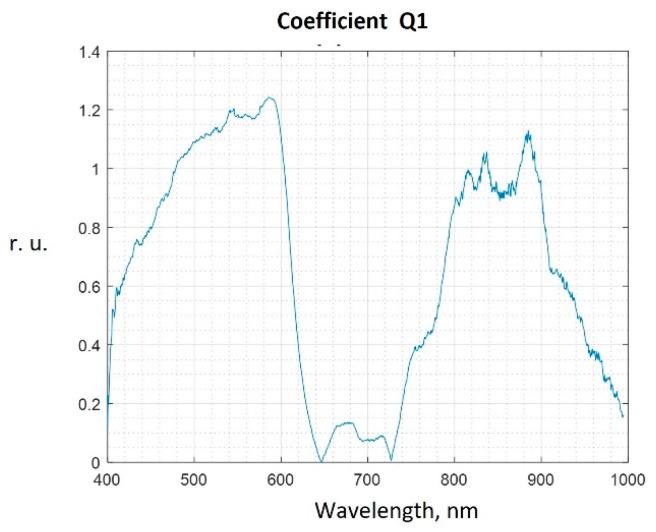
Graph of the Q coefficient for patient No. 1 (areas of hyperkeratosis on the tongue).

Figure 4 shows the values of the Q coefficient calculated by comparing the spectra of hyperkeratosis areas and intact tongue tissues in patient No. 1.

We set the threshold value of the coefficient Qth, starting from which the wavelength subranges are considered informative. Wavelength subranges below the Qth threshold value were not included in the feature vector for constructing the classifier. The informative subranges chosen in this way are used for automatic spectrum classification.

For the cheek tissue spectra, the classifier parameters are analyzed at three Qth values: 1.0, 0.8, and 0.4, four Qth values are chosen for the tongue tissue spectra: 1.2; 1.0; 0.7, and 0.4.

**Figure 5:**
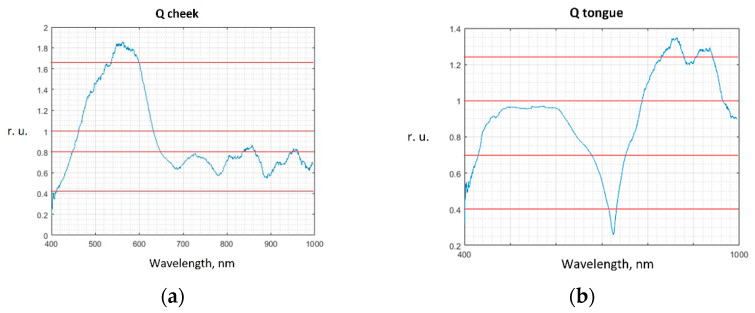
Graph of the total Q coefficient for the tissues of the cheek (**a**) and for the tissues of the tongue (**b**) with hyperkeratosis. Red lines—Qth values.

The values of the Q coefficients obtained from the samples for the cheek and tongue tissues, as well as the selected Qth values, are shown in Figure 5.

As the parameters of the feature vector were used as the input data of the classifier, the areas under the spectrum section are used, limited by the informative subranges of wavelengths Δ*λi* corresponding to the considered current value *Qthi*:

(4) $$S_{n}\left(\Delta \lambda_{i}\right)=\sum_{Q\left(\lambda \right)>Q_{thi}}I\left(\lambda \right)\Delta \lambda_{i},$$

The following classification methods were tested in the study: decision trees (DT), linear discriminant analysis (DA), logistic regression (LR), naive Bayes classifiers (NKB), support vector machines (SVM), and k-nearest neighbors (KNN).

For each type of tissue (epithelium of the buccal mucosa and epithelium of the tongue mucous membrane), we classified them into two classes, which were “intact tissues” and “hyperkeratosis”.

For each classifier method, one of the possible classification results described in Table 1 was observed. Each classification result corresponds to the value of the relative frequency of observation N.

In accordance with the values of the observation relative frequencies, the sensitivity *Se* and the specificity *Sp* of the classification method were calculated using the given selected threshold Qthi:

(5) $$Se=\frac{N_{TP}}{N_{TP}+N_{FN}},\;\;\;\;\;\;Sp=\frac{N_{TN}}{N_{TN}+N_{FP}},$$

The sensitivity and specificity of the classification algorithm for *Qthi* were evaluated. Next, the classification procedure and sensitivity and specificity were repeated for the new *Qthi* value.

We used principal component analysis (PCA) [39,40] to select features that describe 95 percent of the variance of the original data. The spectrum sample was divided into training and test samples using the cross-validation method: for each test series, the average spectra of the “hyperkeratosis” and “intact tissues” classes were randomly divided into five samples, of which one was a test sample, and the remaining four were training samples. Thus, the output parameters of the classifier during each series of tests were calculated as an average value over five training cycles.

In the second stage of the study, the spectra of 101 patients with different lesions of the oral mucosa were classified (Table 2).

Random forest was used as a classifier. Normal and pathological signals were applied to the input of the classifier in various ranges.

## 3. Results and Discussion

In the first stage, we calculated the relative frequencies of hyperkeratosis areas’ automatic detection by tested classifiers. The obtained values of the relative frequencies are presented in Table 3 and Table 4.

**Figure 6:**
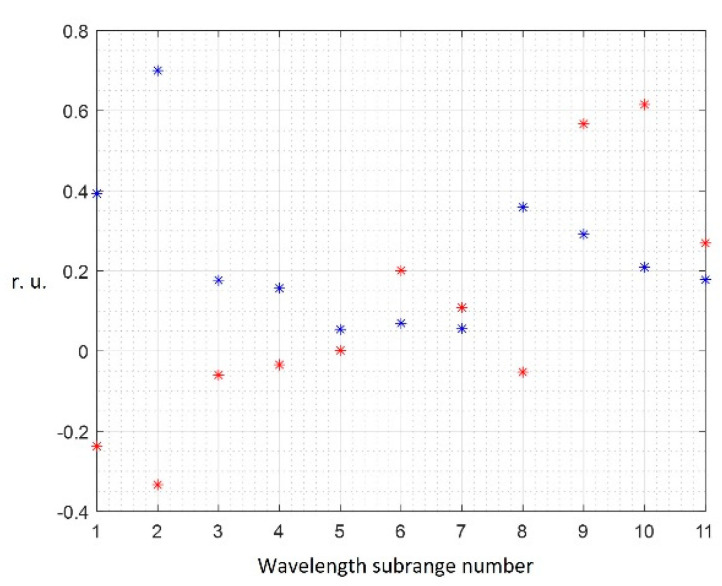
Loading plot Diagram of the first (blue dots) and second (red dots) principal components (buccal mucosa, Qth = 0.4).

Using PCA, it is possible to refine the informative wavelength range when hyperkeratosis is detected on the buccal mucosa. The loading plot in Figure 6 shows the contribution of the numbered wavelength subranges to the first two principal components, which describe 95 percent of the input data variance.

Considering the loading plot, we can conclude that the subranges under the sixth (from 830 to 870 nm) and eighth numbers (from 410 to 450 nm), which make the minimum contribution to the first two principal components, can be excluded from the input data to reduce dimensions of the feature vector.

Thus, the best results in the detection of hyperkeratosis by backscattering spectra were obtained using discriminant analysis in the wavelength range from 450 to 650 nm and from 750 to 1000 nm in case of detection of hyperkeratosis on the buccal mucosa, and when using the k-nearest neighbors method in the wavelength ranges waves from 450 to 830 nm and from 870 to 1000 nm in case of detection of hyperkeratosis on normally non-keratinized areas of the mucous membrane of the tongue.

According to the test results presented in Table 1 and Table 2, the values of sensitivity and specificity for the detection of hyperkeratosis are determined using an automatic classification of backscatter spectra. The results of the calculation of sensitivity and specificity are presented in Table 5.

**Figure 7:**
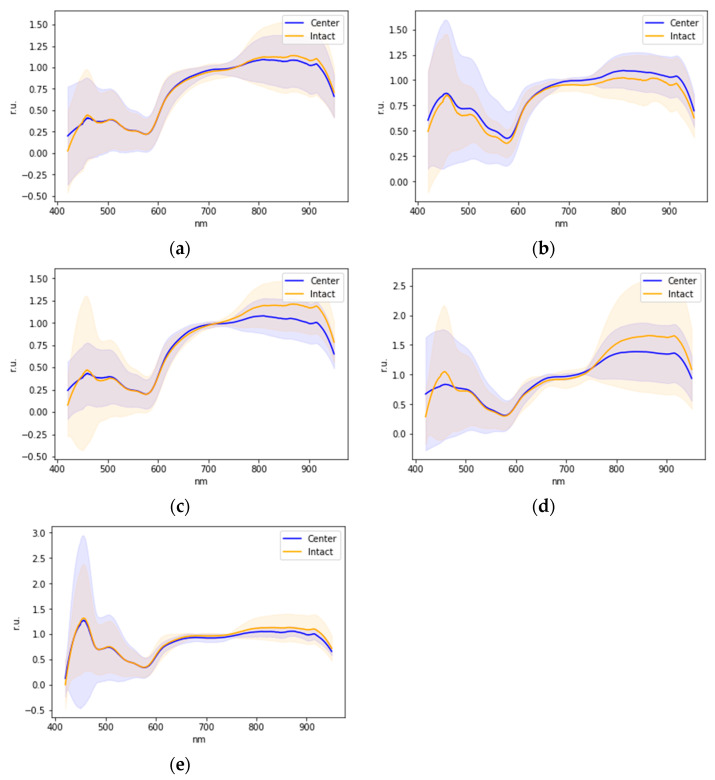
Sample-averaged spectra of different types of lesions: lichen planus (**a**), leukoplakia (**b**), traumatic erosion (**c**), glossitis (**d**), and fibroma (**e**).

During the analysis of the patients’ sample spectra during the second stage of the study (Table 2), the average graphs of the intact and lesion areas’ spectra were plotted for the sample, indicating the dispersion in the sample (Figure 7).

Due to the high degree of dispersion in the data of the intact and lesion areas’ spectra, it follows that the statistical separation of classes is impossible.

Evaluating the initial data without using normalization, it was found that the data of most patients differ by some delta, regardless of which signal amplitude is predominant.

In this regard, the hypothesis of individual distinguishability of the spectra of intact and lesion areas in patients was tested with the help of modeling. To test this hypothesis, a reference signal of the intact area was modeled, with respect to which it will be possible to determine the presence of a lesion in each particular patient. Signal modeling for each patient was carried out by randomly selecting of 15 measurements from 15 initial measurements for the intact area.

**Figure 8:**
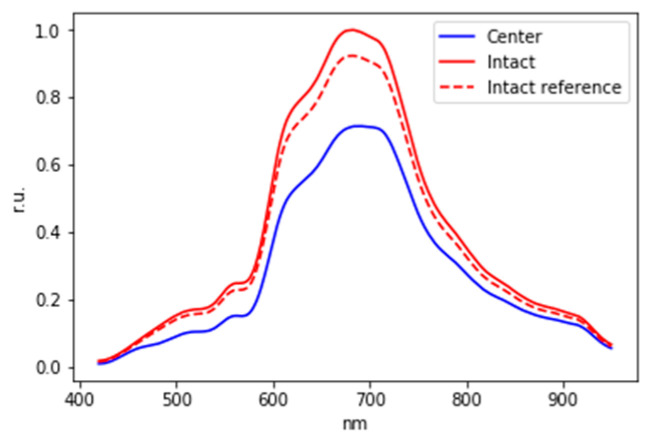
Normalized to the maximum value among three signals: the intact area spectrum, lesion spectrum, and reference intact area spectrum.

To consider the delta for the binary classification of these patients, the initial signals were normalized to the maximum value among three signals (intact, lesion, and reference signal of the intact area). Thus, an example of normalized graphs is shown in Figure 8.

**Figure 9:**
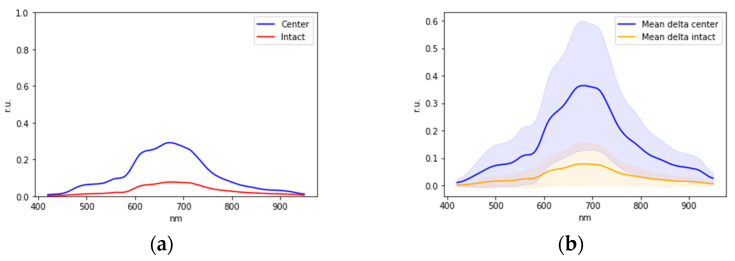
The difference between the norm and the reference signal of the norm and the difference between the intact and the reference intact signal for one of 101 patients in the sample (**a**); mean values and standard deviations of the difference between the intact and lesion areas (**b**).

To build a classifier for each patient, the difference between the intact and the reference signal of the intact area also as the difference between the lesion area and the reference signal of the intact area were calculated. The difference between the spectra for one of the patients, as well as the mean values and standard deviations of the difference between the intact area and lesion area, is shown in Figure 9.

The results of spectra classification accuracy estimation for different wavelength ranges are shown in Table 6. 

Thus, as a result of preliminary in vivo experimental studies, the possibility of automatic detection using DRS of hyperkeratosis areas on the mucosa of the ventral surface of the tongue in the wavelength ranges from 450 to 650 nm and from 750 to 1000 nm and on the buccal mucosa in the wavelength ranges from 450 to 830 nm and from 870 to 1000 nm was shown.

Accounting for individual patient characteristics allows for the automatic detection of lesion areas in oral mucosa using the DRS method in the wavelength range from 500 to 900 nm with an accuracy of at least 75 percent.

As a result of studies of the DRS method, Raman spectroscopy (RS), hyperspectral imaging (HSI), and fluorescence spectroscopy (FS) in the wavelength range from 400 to 1700 nm, published by other authors [31,51,52,53,54,55,56,57,58,59,60], values of accuracy, sensitivity, and accuracy of automatic classification of the spectra of the esophagus, stomach, pancreas, cervical, tongue, buccal, gingiva, oral mucosa, and liver tissue ranging from 79 to 99 percent by machine learning methods were obtained (Table 7).

At the same time, the published results [49,50,51,52,53,54,55,56,57,58,59] were obtained mostly on ex vivo tissue samples. Thus, the classification accuracy of 75% obtained by us under in vivo conditions, which are characterized by less stable registration conditions and greater variability of tissue parameters, shows the promise of further studies of the DRS method in vivo. The informative wavelength range of the spectra classification from 500 to 900 nm is consistent with the biophysical explanation of the changes in the absorbing and scattering properties of mucosal tissues during pathological changes. It is supposed to improve the values of classification accuracy in the course of further studies by increasing the sample sizes of registered reference spectra of normal tissue areas, making a more detailed choice of informative wavelengths, particularly using PCA, combining the results of spectral analysis with the results of other studies, particularly fluorescence, hyperspectral imaging, Raman spectroscopy, using deep learning methods for spectra classification.

During further studies, we plan to test the DRS method on a larger sample of data, with the registration of reference normal spectra, to achieve better classification accuracy, with a greater variety of forms and localization of mucosal lesions. We also plan to conduct a more detailed study of the correlation between changes in DRS spectra and histopathological changes in oral mucosa, including specifics of lesions caused by COVID-19. This will help to determine the diagnostic capabilities of the DRS in detecting early stages, including preclinical stages of precancerous lesions of the oral mucosa, to verify the results of the DRS using the results of immunohistochemical studies, as well as using the results of other instrumental methods, to determine the technical parameters of the hardware-software complex for DRS of the oral mucosa in vivo and the method of its clinical application.

## Figures and Tables

**Table 1:** Classification results.

Classification Result	Relative Frequency	Interpretation
True positive	N_TP_	The spectrum of the “hyperkeratosis” class is correctly classified as the spectrum of The “hyperkeratosis” class
False positive	N_FP_	“intact area” class spectrum is falsely classified as “hyperkeratosis” class spectrum
True negative	N_TN_	The spectrum of the “intact area” class is correctly classified as the spectrum of the “intact area” class
False negative	N_FN_	“hyperkeratosis” class spectrum is falsely classified as “intact area” class spectrum

**Table 2:** Characteristics of lesion spectrum samples.

No.	Lesion Type	Number of Patients
1	Lichen planus	25
2	Leukoplakia	9
3	Traumatic erosion	16
4	Glossitis	6
5	Fibroma	4
6	Unmarked lesions	41

**Table 3:** The Results of Automatic Hyperkeratosis Areas Detection on the Mucous Membrane of the Tongue.

Method	Relative Frequencies of Hyperkeratosis Areas Detection on the Mucous Membrane of the Tongue							
	N_TP_		N_FN_		N_FP_		N_TP_	
	w/o PCA	PCA	w/o PCA	PCA	w/o PCA	PCA	w/o PCA	PCA
	***Q_th_* = 1.2**							
DT	0.69 ± 0.06	0.73 ± 0.03	0.40 ± 0.05	0.38 ± 0.08	0.31 ± 0.06	0.27 ± 0.03	0.60 ± 0.05	0.63 ± 0.08
DA	0.81 ± 0.03	0.81 ± 0.03	0.33 ± 0.06	0.40 ± 0.05	0.19 ± 0.03	0.19 ± 0.03	0.68 ± 0.06	0.60 ± 0.05
LR	0.80 ± 0.05	0.79 ± 0.01	0.38 ± 0.01	0.40 ± 0.05	0.20 ± 0.05	0.21 ± 0.01	0.63 ± 0.01	0.60 ± 0.05
NKB	0.79 ± 0.01	0.84 ± 0.05	0.33 ± 0.06	0.48 ± 0.15	0.21 ± 0.01	0.16 ± 0.05	0.68 ± 0.06	0.53 ± 0.15
SVM	0.87 ± 0.05	0.86 ± 0.05	0.38 ± 0.06	0.43 ± 0.06	0.13 ± 0.05	0.14 ± 0.05	0.63 ± 0.11	0.58 ± 0.06
kNN	0.90 ± 0.07	0.96 ± 0.09	0.45 ± 0.06	0.90 ± 0.20	0.10 ± 0.07	0.04 ± 0.09	0.55 ± 0.13	0.10 ± 0.09
	***Q_th_* = 1.0**							
DT	0.79 ± 0.01	0.71 ± 0.01	0.25 ± 0.01	0.28 ± 0.05	0.21 ± 0.01	0.29 ± 0.01	0.75 ± 0.01	0.73 ± 0.05
DA	0.79 ± 0.01	0.80 ± 0.03	0.28 ± 0.09	0.45 ± 0.06	0.21 ± 0.01	0.20 ± 0.03	0.73 ± 0.09	0.55 ± 0.06
LR	0.79 ± 0.05	0.79 ± 0.01	0.55 ± 0.06	0.45 ± 0.06	0.21 ± 0.05	0.21 ± 0.01	0.45 ± 0.06	0.55 ± 0.06
NKB	0.79 ± 0.01	0.81 ± 0.06	0.25 ± 0.08	0.45 ± 0.10	0.21 ± 0.01	0.19 ± 0.06	0.75 ± 0.08	0.55 ± 0.10
SVM	0.81 ± 0.07	0.84 ± 0.07	0.40 ± 0.09	0.48 ± 0.15	0.19 ± 0.07	0.16 ± 0.07	0.60 ± 0.09	0.53 ± 0.15
kNN	0.89 ± 0.10	0.89 ± 0.10	0.65 ± 0.22	0.65 ± 0.24	0.11 ± 0.10	0.11 ± 0.10	0.35 ± 0.22	0.35 ± 0.24
	***Q_th_* = 0.7**							
DT	0.84 ± 0.06	0.90 ± 0.06	0.23 ± 0.09	0.88 ± 0.14	0.16 ± 0.03	0.10 ± 0.06	0.78 ± 0.09	0.13 ± 0.10
DA	0.80 ± 0.05	0.99 ± 0.03	0.18 ± 0.06	0.98 ± 0.05	0.20 ± 0.05	0.01 ± 0.03	0.83 ± 0.06	0.03 ± 0.03
LR	0.74 ± 0.09	0.97 ± 0.03	0.63 ± 0.14	0.98 ± 0.05	0.26 ± 0.09	0.03 ± 0.03	0.38 ± 0.14	0.03 ± 0.03
NKB	0.81 ± 0.06	0.91 ± 0.05	0.23 ± 0.15	0.90 ± 0.09	0.19 ± 0.06	0.09 ± 0.05	0.78 ± 0.15	0.10 ± 0.09
SVM	0.93 ± 0.06	1.00 ± 0.01	0.38 ± 0.11	0.98 ± 0.05	0.07 ± 0.06	0.00	0.63 ± 0.11	0.03 ± 0.03
kNN	0.90 ± 0.07	1.00 ± 0.01	0.38 ± 0.26	1.00 ± 0.01	0.10 ± 0.07	0.00	0.63 ± 0.26	0.00
	***Q_th_* = 0.4**							
DT	0.84 ± 0.03	0.81 ± 0.03	0.28 ± 0.05	0.85 ± 0.15	0.16 ± 0.03	0.19 ± 0.03	0.73 ± 0.05	0.15 ± 0.09
DA	0.74 ± 0.06	0.89 ± 0.06	0.13 ± 0.01	1.00 ± 0.01	0.26 ± 0.06	0.11 ± 0.06	0.88 ± 0.01	0.00
LR	0.64 ± 0.08	0.87 ± 0.07	0.70 ± 0.13	1.00 ± 0.01	0.36 ± 0.08	0.13 ± 0.07	0.30 ± 0.13	0.00
NKB	0.81 ± 0.03	0.90 ± 0.06	0.40 ± 0.05	0.88 ± 0.11	0.19 ± 0.03	0.10 ± 0.06	0.60 ± 0.05	0.13 ± 0.11
SVM	0.90 ± 0.07	1.00 ± 0.01	0.50 ± 0.08	1.00 ± 0.01	0.10 ± 0.07	0.00	0.50 ± 0.08	0.00
kNN	0.94 ± 0.07	1.00 ± 0.01	0.50 ± 0.11	1.00 ± 0.01	0.06 ± 0.07	0.00	0.50 ± 0.11	0.00

**Table 4:** The Results of Automatic Hyperkeratosis Areas Detection on the Buccal Mucosa.

Method	Relative Frequencies of Hyperkeratosis Areas Detection on the Mucous Membrane of the Tongue							
	N_TP_		N_FN_		N_FP_		N_TP_	
	w/o PCA	PCA	w/o PCA	PCA	w/o PCA	PCA	w/o PCA	PCA
	***Q_th_* = 1.0**							
DT	0.37 ± 0.07	0.50 ± 0.01	0.11 ± 0.06	0.00	0.63 ± 0.07	0.50 ± 0.01	0.89 ± 0.06	1.00 ± 0.01
DA	0.50 ± 0.01	0.50 ± 0.01	0.14 ± 0.01	0.17 ± 0.06	0.50 ± 0.01	0.50 ± 0.01	0.86 ± 0.01	0.83 ± 0.06
LR	0.57 ± 0.08	0.50 ± 0.01	0.34 ± 0.07	0.23 ± 0.07	0.43 ± 0.08	0.50 ± 0.01	0.66 ± 0.07	0.77 ± 0.07
NKB	0.50 ± 0.01	0.47 ± 0.07	0.23 ± 0.07	0.20 ± 0.07	0.50 ± 0.01	0.53 ± 0.07	0.77 ± 0.07	0.80 ± 0.07
SVM	0.70 ± 0.12	0.47 ± 0.07	0.06 ± 0.11	0.03 ± 0.06	0.30 ± 0.12	0.53 ± 0.07	0.94 ± 0.11	0.97 ± 0.06
kNN	0.77 ± 0.23	0.87 ± 0.12	0.20 ± 0.19	0.46 ± 0.14	0.23 ± 0.21	0.13 ± 0.12	0.80 ± 0.19	0.54 ± 0.14
	***Q_th_* = 0.8**							
DT	0.77 ± 0.13	0.43 ± 0.13	0.14 ± 0.09	0.03 ± 0.03	0.23 ± 0.13	0.57 ± 0.13	0.86 ± 0.09	0.97 ± 0.06
DA	0.57 ± 0.17	0.43 ± 0.13	0.20 ± 0.07	0.14 ± 0.01	0.43 ± 0.17	0.57 ± 0.13	0.80 ± 0.07	0.86 ± 0.01
LR	0.43 ± 0.13	0.47 ± 0.07	0.34 ± 0.15	0.23 ± 0.07	0.57 ± 0.13	0.53 ± 0.07	0.66 ± 0.15	0.77 ± 0.07
NKB	0.67 ± 0.11	0.47 ± 0.07	0.26 ± 0.06	0.20 ± 0.07	0.33 ± 0.11	0.53 ± 0.07	0.74 ± 0.06	0.80 ± 0.07
SVM	0.80 ± 0.07	0.43 ± 0.08	0.00	0.06 ± 0.11	0.20 ± 0.07	0.57 ± 0.08	1.00 ± 0.01	0.94 ± 0.11
kNN	0.80 ± 0.16	0.63 ± 0.22	0.11 ± 0.06	0.34 ± 0.11	0.20 ± 0.16	0.37 ± 0.22	0.89 ± 0.06	0.66 ± 0.11
	***Q_th_* = 0.4**							
DT	0.67 ± 0.11	0.27 ± 0.08	0.20 ± 0.11	0.00	0.33 ± 0.11	0.73 ± 0.08	0.80 ± 0.11	1.00 ± 0.01
DA	0.70 ± 0.12	0.97 ± 0.07	0.26 ± 0.06	0.20 ± 0.15	0.30 ± 0.12	0.03 ± 0.07	0.74 ± 0.06	0.80 ± 0.15
LR	0.67 ± 0.21	0.70 ± 0.07	0.60 ± 0.11	0.14 ± 0.01	0.33 ± 0.21	0.30 ± 0.07	0.40 ± 0.11	0.86 ± 0.01
NKB	0.77 ± 0.08	0.97 ± 0.07	0.23 ± 0.07	0.20 ± 0.15	0.23 ± 0.08	0.03 ± 0.01	0.77 ± 0.07	0.80 ± 0.15
SVM	0.90 ± 0.13	0.93 ± 0.08	0.00	0.00	0.10 ± 0.09	0.07 ± 0.05	1.00 ± 0.01	1.00 ± 0.01
kNN	0.87 ± 0.07	0.97 ± 0.07	0.06 ± 0.05	0.00	0.13 ± 0.07	0.03 ± 0.01	0.94 ± 0.07	1.00 ± 0.01

**Table 5:** The results of the calculation of sensitivity and specificity.

Parameter	The Mucous Membrane of the Ventrolateral Surface of the Tongue	The Mucous Membrane of the Cheek
Sensitivity	0.83 ± 0.17	1.00 ± 0.00
Specificity	0.80 ± 0.17	0.97 ± 0.20

**Table 6:** Accuracy of pathology detection in a patient.

Wavelength Range, nm	Classification Accuracy, %	Wavelength Range, nm	Classification Accuracy, %
500–900	75.00	600–800	75.62
500–850	75.50	600–750	75.64
500–800	75.00	600–701	75.73
500–750	74.75	650–900	75.38
500–701	74.80	650–850	75.29
550–900	75.00	650–800	75.17
550–850	75.14	650–750	74.95
550–800	75.12	650–701	74.55
550–750	75.11	700–900	74.33
550–701	75.00	700–800	74.22
600–900	75.27	700–750	74.12
600–850	75.42	700–701	73.36
700–850	74.27		

**Table 7:** Performance parameters of the spectra classification evaluation.

Method	Tissue Type	Wavelength Range, nm	Specificity, %	Sensitivity, %	Accuracy, %	Reference
DRS in vivo	Oral mucosa	500–900	-	-	75	Current study
DRS ex vivo	Oral mucosa	400–1700	89	82	86	[51]
DRS ex vivo	Esophagus mucosa; stomach mucosa	400–1000	-	-	96—for esophagus mucosa; 94—for stomach mucosa	[52]
DRS ex vivo	Colorectal mucosa	400–1000	91	92	91	[53]
DRS ex vivo	Liver tissue	450–1550	99	100	-	[54]
HSI ex vivo	Esophagus tissue	400–750	-	-	85–95	[55]
RS ex vivo	Pancreatic tissue	400–700	91	82	-	[56]
RS in vivo	Cervical tissue	355–655	79	78	-	[57]
DRS ex vivo	Tongue tissue	400–1000	84	80	82	[58]
FS ex vivo	Oral mucosa	325	100	93–97	96–98	[59]
RS in vivo	Tongue tissue/Buccal mucosa/Gingiva tissue	532	92–100/79–84/75–92	63–72/93/91–100	79–88/85–88/87–91	[31]
RS ex vivo	Tongue tissue	785	99	99	-	[60]

## Data Availability

The data presented in this study are available on request from the corresponding author. The data are not publicly available due to ethical restrictions.
